# Implementation of the Ironbark falls prevention program: a mixed methods process evaluation with Aboriginal communities

**DOI:** 10.1093/ageing/afag184

**Published:** 2026-06-22

**Authors:** Grace McKeon, Zarayn Knight, Rebecca Ivers, Julieann Coombes, Vanessa Jessup, Kim Delbaere, Catherine Sherrington, Anne Tiedemann, Anne-Marie Hill, Leanne McCarthy, Bronte Haynes, Courtney Ryder, Rona Macniven, Ironbark Investigator Group, Kathie Clapham, Lindy Clemson, Robert Cumming, Margaret Gidgup, Josephine Gwynn, Kirsten Howard, Kate Hunter, Stephen Jan, Marion Kickett, Lisa Keay, Cassia Lindsay, Tamara Mackean, Kris Rogers, Judy Simpson, Amy Townsend

**Affiliations:** Institute of Physical Activity and Nutrition, School of Exercise and Nutrition Sciences, Deakin University, Melbourne, Victoria, Australia; Discipline of Psychiatry and Mental Health, School of Clinical Medicine, University of New South Wales, Sydney, New South Wales, Australia; School of Population Health, University of New South Wales, Sydney, New South Wales, Australia; School of Population Health, University of New South Wales, Sydney, New South Wales, Australia; The George Institute for Global Health, Sydney, New South Wales, Australia; The George Institute for Global Health, Sydney, New South Wales, Australia; WA Centre for Health and Ageing, The University of Western Australia, Perth, Australia; School of Health Sciences, Health Translation Hub, University of New South Wales, Randwick, New South Wales 2031, Australia; Falls, Balance and Injury Research Centre, Neuroscience Research Australia, Randwick, New South Wales 2031, Australia; Institute for Musculoskeletal Health, Sydney Local Health District, Camperdown, New South Wales, Australia; School of Public Health, The University of Sydney Faculty of Medicine and Health, Sydney, New South Wales, Australia; Institute for Musculoskeletal Health, Sydney Local Health District, Camperdown, New South Wales, Australia; School of Public Health, The University of Sydney Faculty of Medicine and Health, Sydney, New South Wales, Australia; School of Allied Health, The University of Western Australia, 35 Stirling Hwy, Crawley, Perth, Western Australia 6009, Australia; WA Centre for Health and Ageing, Perth 6009, Australia; School of Population Health, University of New South Wales, Sydney, New South Wales, Australia; Ngarruwan Ngadju First Peoples Health and Wellbeing Research Center, University of Wollongong, Wollongong, New South Wales, Australia; School of Population Health, University of New South Wales, Sydney, New South Wales, Australia; College of Medicine and Public Health, Flinders University, Adelaide, South Australia, Australia; School of Population Health, University of New South Wales, Sydney, New South Wales, Australia

**Keywords:** Aboriginal and Torres Strait Islander peoples, older adults, falls prevention, exercise, implementation, qualitative research

## Abstract

**Background:**

Falls are a leading cause of injury and loss of independence among older Aboriginal people. The Ironbark program is a culturally co-designed, community-based fall prevention initiative, incorporating exercises and Yarning circles, a culturally grounded form of group discussion. This paper presents a process evaluation of the program, examining fidelity and key factors influencing implementation across settings.

**Methods:**

Sixteen sites across two Australian states implemented the Ironbark Standing Strong and Tall program. Process data were collected from 15 sites through structured session observations and semi-structured interviews with exercise facilitators. Fidelity and dose were assessed quantitatively, with adherence scored using a summative checklist score. Qualitative data were thematically analysed and mapped onto the Consolidated Framework for Implementation Research (CFIR).

**Results:**

The program was delivered with high fidelity to 117 participants with median adherence scores consistently above 80%. Sessions were implemented as planned, with strong participant engagement in both exercise and Yarning components. Key facilitators included Aboriginal leadership, trusted site managers, program flexibility and supportive service structures. Challenges included managing varying physical abilities, maintaining engagement over time and external disruptions (e.g. Coronavirus -19 pandemic, weather). Content repetition was noted as a barrier to sustained attendance by participants in later stages. Facilitators recommended additional training, mid-program assessments and access to site-collected data. Sustainability was linked to workforce capacity and securing ongoing funding, particularly to support Aboriginal staff and transport.

**Conclusion:**

This process evaluation indicated that a culturally-relevant fall prevention program for older Aboriginal people can be implemented with fidelity across community settings. Findings provide practical guidance for exercise facilitators and on sustaining and expanding the Ironbark program and inform broader efforts to embed culturally safe, community-led health programs in routine practice.

## Key points

- The Ironbark program fall prevention initiative for older Aboriginal people was implemented with high fidelity across 15 sites.
- Aboriginal leadership, trusted site managers and program flexibility were important facilitators of program delivery.
- Challenges included managing diverse physical abilities, repetition in content delivery and external disruptions like natural disasters and COVID-19.
- Sustainability was perceived to depend on dedicated funding, recognition of cultural identity, community trust and a skilled local workforce.

## Introduction

Falls and fall-related injury are a growing health concern for older Aboriginal people [1, 2]. In Australia, falls are the leading cause of hospitalised injuries and injury-related deaths among older adults, accounting for 77% of all injury hospitalisations and 71% of injury-related deaths in this age group [3]. Severe fall injuries in older people are associated with reduced mobility, decreased quality of life, loss of independence and increased probability of requiring residential care [4, 5]. However, the burden of falls is not equally distributed. Older Aboriginal people experience higher rates of falls [1] and their consequences, which may include separation from home country, community and culture.

Maintaining health and wellbeing to remain independent and contribute to family and community is a priority for older Aboriginal people [6]. There is robust evidence showing that exercise that incorporates balance and strength activities can reduce the rate of falls by up to 23% [7]. In addition, culturally safe physical activity programs- defined as those that respect Aboriginal peoples’ cultural values, strengths and differences and actively address racism and power imbalances [8] can improve social and emotional wellbeing for First Nations People [9].

The Ironbark Standing Strong and Tall program is a group-based fall prevention program for Aboriginal people aged 45 years and over. The program was co-designed and piloted from 2014 to 2016 in collaboration with Aboriginal communities in New South Wales (NSW). It was implemented over a period of 3–6 months with six NSW community sites and 98 participants [10] and over a 12–24 month period with two groups of 52 participants in total in Western Australia (WA) [11]. The program is evidence-based, and includes supervised weekly balance and strength sessions, a home exercise program and Yarning circles on falls risk and prevention strategies [12]. Pilot evaluation findings demonstrated improvements in strength and balance, reduced body mass index (BMI) and strong community acceptance and program engagement [10, 11]. Following the pilot study, an implementation study was conducted from 2021 to 2023, comparing the *Ironbark Standing Strong and Tall* program with a healthy community group-based social program. In the context of pandemic-related disruptions, this implementation study focused on implementation processes and physical outcomes, which are reported elsewhere.

Exercise professionals are well equipped to prescribe fall prevention exercises and manage falls in community-dwelling older adults, particularly for those who are at high risk of falls and have a range of limitations [13, 14]. There has been research examining the perspectives of key stakeholders [15] and community members [10] involved in implementing Ironbark during the pilot phase; however, limited research exists on the implementation of falls prevention programs in Aboriginal communities from the perspectives of those delivering them. There is also limited research on the implementation or scale-up of physical activity interventions [16]. It is therefore important to shift the focus from small-scale, tightly controlled interventions to evaluating the implementation and translation of community-based physical activity programs [17, 18].

This study presents a nested process evaluation of the Ironbark program, developed by Aboriginal and non-Aboriginal study investigators and project staff [19]. Process evaluations examine the implementation aspects of a program or service, how programs work in practice and whether they were delivered as intended and the quantity, or dose of implementation [20]. Therefore, using a mixed-method, multi-site evaluation design, this study addressed the following two research questions:

1) To what extent was the Ironbark program implemented as intended (fidelity and dose)?
2) What were the experiences of the exercise program facilitators and what factors influenced how the program was implemented?

## Methods

### Study design and setting

This study employed a mixed-method, multi-site evaluation design utilising data collected through semi-structured interviews with facilitators, site visit observations and facilitator self-report implementation checklists from 15 sites delivering the 12-month *Ironbark Standing Strong and Tall* exercise program, across two Australian states; NSW and WA. In total, 117 participants took part in the Ironbark Standing Strong and Tall program and completed baseline and follow-up surveys.

### Ironbark intervention components

The Ironbark Standing Strong and Tall program is a group-based fall prevention program comprising a 45-minute, once-weekly balance and strength exercise session, based on Otago Exercise Program [21], alongside a Yarning circle focused on falls risk and prevention strategy topics. Light catering (e.g. morning tea) was provided. Participants received a booklet with home exercise guidance and were encouraged to complete 10–15 minutes of exercises daily. The exercises were delivered by a trained facilitator, such as a physiotherapist, exercise physiologist, or personal trainer. Yarning circles were facilitated by the site manager or exercise professional, depending on the site preference.

### Ironbark implementation strategies

The research team provided approximately four hours of program-specific training to each site. During these sessions, an exercise physiologist or physiotherapist guided and demonstrated the falls prevention exercises using the manual, while an Aboriginal researcher provided training on conducting the Yarning circle. An optional one-day falls prevention training session was also offered by an external provider. Program materials, including facilitator training manuals and participant exercise booklets, were supplied to all sites to support consistent delivery. The site managers were engaged to facilitate program coordination, support integration within the service and adapt the sessions based on local context, cultural safety considerations and participant preferences, for example, determining group composition (e.g. mixed-gender or gender specific groups).

### Ethics

Ethics approval was provided by the Aboriginal Health and Medical Research Council of NSW (reference 1401/18), and WA Aboriginal Health Ethics Committee (reference 962) and noted by the University of New South Wales and University of Western Australia Human Research Ethics Committee. Written informed consent was obtained from all participants.

### Participants

Ten of the interviewed facilitators were women and four were men. Years of experience as an exercise professional ranged from 2 to 30 years (median = 10 years), noting that the same facilitator was used at three sites in Western Australia and at two sites in New South Wales, so treated as one person when calculating the median. Six facilitators were physiotherapists, seven were exercise physiologists and 1 was a Certificate-IV personal trainer. All facilitators had experience working in falls prevention with older adults, while 12 had experience working with Aboriginal people and one facilitator identified as Aboriginal.

### Data collection

#### Fidelity and dose assessment

Program delivery and adherence were assessed via implementation checklists, which were used within the first two months of program delivery and at 6 and 9 months. Program adherence (fidelity) is defined as a measure of the quality of program implementation [22]. A checklist and coaching tool were used to assess the quality of delivery, including the concepts, content, activities, engagement and delivery strategies; the development and protocol for this tool have been described previously [19]. The tool (Appendix 3) was developed by the research team, including Indigenous researchers was informed by a theory-of-change framework; however, formal reliability and validity testing have not been conducted. It included an open-ended comments section to document details on broader relevant aspects such as participant and facilitator interaction, facility and equipment suitability and cultural appropriateness. The project officer completed the checklist while conducting a site visit and observing a session. They also provided feedback and coaching for the exercise facilitator.

**Table 1:** Program delivery scores.

**Component, *N* (%)**	**8 weeks (*n* = 12)**	**6 months (*n* = 14)**	**9 months (*n* = 9)**
Delivered to plan			
Yes	12 (100)	14 (100)	7(78)
No	0(0)	0(0)	2(22)
Duration of Yarn (min)			
<30	0(0)	2(14)	1(11)
30–45	2(17)	5(36)	2(22)
45–60	6(50)	1(7)	2(22)
60+	2(17)	4(29)	4(44)
N/A	2(17)	0(0)	0(0)
Participant involvement- Yarn			
5 (very relevant)	7(58)	11(79)	6(63)
4	3(25)	3(21)	1(13)
3	1(8)	0(0)	1(13)
N/A	1(8)	0(0)	0(0)
Relevance of Yarn			
5 (very relevant)	7(58)	8(57)	5(55)
4	3(25)	6(43)	2(22)
3	1(8)	0(0)	1(11)
N/A	1(8)	0(0)	0(0)
Duration of exercise (min)			
<30	1(8)	0(0)	0(0)
30–45	10(83)	9(64)	5(55)
45–60	1(8)	5(36)	4(44)
Participant involvement - exercise			
5	7(58)	10(71)	6(67)
4	3(25)	4(29)	2(22)
3	2(17)	0(0)	1(11)
Modified session to needs			
Yes	10(83)	14(100)	8(89)
No	2(17)	0(0)	0(0)
N/A	0(0)	0(0)	1(11)
Corrected participant exercise technique			
Yes	11(92)	12(86)	7(78)
No	1(8)	1(7)	0(0)
Unsure or N/A	0(0)	1(7)	2(22)
Importance of equipment			
5 (Very Important)	2(17)	4(29)	3(33)
4	5(42)	6(43)	1(11)
3	0(0)	1(7)	1(11)
2	1(8)	1(7)	1(11)
1	4(33)	0(0)	1(11)
Reminder to complete home exercises			
Yes	8(67)	12(86)	7(78)
No	3(33)	1(7)	2(22)
Unsure	1(8)	0(0)	0(0)
Appropriate exercise for levels and abilities			
Yes	10(83)	12(86)	8(89)
No	0(0)	0(0)	0(0)
Unsure	2(17)	2(14)	0(0)
N/A	0(0)	0(0)	1(11)

*Note: Percentages may not sum to 100% due to rounding or missing data.*

Program delivery (dose delivered) is a measure of the extent to which all the intended components of the *Ironbark: Standing Strong and Tall* program were delivered to participants [22]. A questionnaire (Appendix 4) was completed at 2, 6 and 9 months by each exercise facilitator, capturing sessions delivered as plan, time spent in the exercise and Yarning components, participant involvement, as well as an open-ended comments. It was initially developed by Aboriginal and non-Aboriginal researchers, with community input, during the Ironbark program development and pilot stage and was updated for the implementation trial [19].

#### Semi-structured interviews

Semi-structured interviews were conducted with the exercise facilitators at the conclusion of the program (between 9 and 12 months) either in person or online via Microsoft Teams. Detailed information on the exercise facilitator experiences and factors influencing implementation of the program was examined. The interview questions (Appendix 1) were based on the five key identified implementation outcomes for the scale-up of physical activity interventions; adoption, dose delivered, reach, fidelity and sustainability [23]. Members of the research team facilitated the interviews (G.M., L.M., V.J. and R.M.), which lasted approximately 30 minutes each.

### Data analysis

A convergent, embedded mixed methods design was used, including triangulation of quantitative and qualitative data [24] guided by Indigenous ways of knowing, being and doing [25] led by authors G.M., a non-Indigenous exercise researcher and Z.K., an Aboriginal project officer. In practice, this involved the use of yarning-informed qualitative approaches, prioritisation of Indigenous voices and interpretation of qualitative findings through a decolonising lens led by an Indigenous researcher.

The analysis was sequential and transformative, meaning the quantitative and qualitative data were collected sequentially, but analysed together [24].

#### Quantitative

The level of program adherence was measured through a summative score from the program checklist and coaching tool (yes = 1; no = 0). Higher checklist scores indicated higher program fidelity. A percentage was calculated based on the max score of 22. Descriptive statistics were used to analyse dosage. Higher scores indicated participants received a higher level of program delivery (i.e. more components of the intervention were delivered within sessions). Open-ended comments were analysed using a content analysis.

#### Qualitative and framework mapping

Firstly, qualitative interviews were transcribed by a data transcription service. Following Braun and Clarkes steps of reflexive thematic analysis, two authors (Z.K. and G.M.) became familiar with the interviews after multiple reads of the transcripts. Secondly, G.M. coded the interviews and Z.K. independently coded 30%, to support reflexive dialogue, code refinement and analytical rigour rather than to establish inter-rater reliability. An inductive thematic analysis was used to identify new ‘candidate’ themes by combining similar codes to create major categories using a thematic map. The themes were then reviewed, checked against the data and ‘candidate’ theme names were provided, clearly reflecting the meaning of each. A decolonising lens was applied to the analysis through privileging Aboriginal voices, knowledge and practices [26].

Final themes were deductively mapped to the Consolidated Framework for Implementation Research (CFIR) [27]. The CFIR is a comprehensive framework that combines constructs from existing theories to facilitate the translation of research findings into practice, primarily within the healthcare sector. It comprises different constructs, grouped into five domains—intervention characteristics, individual characteristics, inner setting, outer setting and process, which can be used to help understand the factors that influence implementation.

## Results

In total, 16 sites delivered the Ironbark Standing Strong and Tall program. Process evaluation data were collected from 15 sites (9 from NSW and 6 from WA). At the one additional site, the program was delivered intermittently due to low participant numbers, and therefore, no process data were collected. Interviews were conducted with the exercise facilitators from 13 out of the 15 sites who agreed to participate. A summary of the completed and missing data is provided in the Supplementary Material (Appendix 2).

### Research question 1: Program fidelity and delivery

#### Checklist and coaching tool (program adherence)

Total implementation scores at 8 weeks across 13 sites ranged from 50% to 100%, and the median score was 19 out of 22 (IQ = 5). At the 6-month time point, scores ranged from 64% to 100% across 14 sites, and the median score was 18.5 (IQ = 3.75). At 9 months, scores ranged from 41% -100% across 9 sites and the median score was 21 (IQ = 4). While there were large ranges, median scores across all 3 times points were >18 (>80%).

To provide context for the observed variability in implementation scores, open-text responses from the checklist and coaching tool were examined. These responses described several factors associated with low adherence during some sessions, including interruptions (e.g. school visits) and transport issues as reasons for low adherence. Despite this, the overall mood during sessions was described as positive, with frequent mentions of laughter, happiness and respect. For example, for example describing the mood as ‘calm, participants engaged, quiet, smiling and laughing,’ or ‘relaxed but focused, happy.’

The use of positive cultural expressions in sessions such as ‘Deadly’ (meaning excellent or fantastic) and ‘Aunty’ (a respectful term for an Elder) was noted. Several responses noted a strong sense of community and that participants arrived early to engage in Yarning sessions with each. However, some barriers were reported, particularly related to mask usage during the COVID-19 pandemic, which affected communication and comfort levels. The type of facilities varied across sites, ranging from outdoor spaces, community centres and smaller boardrooms.

#### Program delivery (dose delivered)

Nearly all sessions were delivered as planned (Table 1), with exceptions at the nine-month mark due to speaker illness and a temporary venue change. At both 6 and 9 months, all exercise sessions lasted at least 30 minutes, though Yarning session durations varied. The perceived importance of exercise equipment varied by site, ranging from high to low across timepoints. Over 80% of facilitators adapted sessions to participant needs and more than 75% corrected exercise technique at each time point.

**Table 2:** Demographic characteristics of interviewed facilitators.

**Site**	**Setting**	**Gender of facilitator**	**Qualification**	**Years of experience**
3	Regional, NSW	W	Exercise physiologist	3
4^b^	Regional, NSW	W	Physiotherapist	25
7^a^	Urban, WA	W	Physiotherapist	30
9^b^	Regional, NSW	W	Physiotherapist	25
10	Regional, NSW	M	Exercise physiologist	10
11^a^	Urban, WA	W	Physiotherapist	30
12^a^	Urban, WA	W	Physiotherapist	30
13	Urban NSW	W	Exercise physiologist & dietitian	14
14	Regional, NSW	W	Exercise physiologist	25
18	Urban, WA	W	Physiotherapist	8
19	Urban, NSW	W	Personal trainer- Cert III and IV	2
20	Regional, NSW	M	Exercise physiologist	6
21a	Regional, NSW	M	Exercise physiologist	10
21b	Urban, NSW	M	Exercise physiologist	2.5

Notes: M = man, W = woman; ^a^ = same participant facilitating different groups; ^b^ = same participant facilitating different groups.

### Research question 2: experiences of the facilitators and factors that influenced implementation

**Figure 1:**
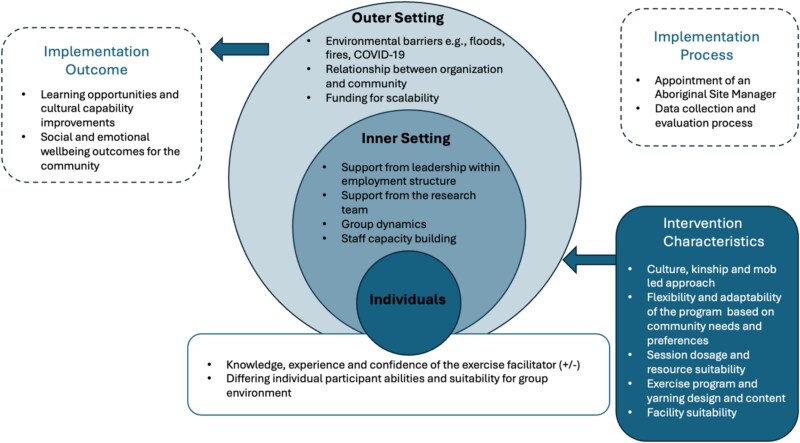
Ironbark standing strong and tall implementation themes mapped to the CFIR framework.

Eighteen themes/constructs were identified and mapped onto the CFIR domains, as shown in Figure 1. Themes are categorised below according to the CFIR domains. The demographic characteristics are shown in Table 2.

### Individual characteristics

Two themes were identified under individual characteristics: (i) the knowledge, experience and confidence of the exercise facilitator, and (ii) the differing individual participant abilities and suitability for the group environment.

Overall, facilitators spoke of having a high level of prior experience and confidence in delivering the exercise component, as the majority were university-trained exercise professionals. Facilitators with less experience or confidence in delivering falls prevention programs or group-based exercise programs appreciated the opportunity to participate in the one-day falls prevention training course. Facilitators described having less experience and confidence regarding the Yarning component, which was commonly linked to nervousness about facilitating Yarning in a culturally sensitive way, particularly when they had not previously facilitated such discussions. This was more frequently reported by facilitators not employed through Aboriginal Medical Services or Aboriginal-led organisations. Opinions varied on whether additional cultural capability training would have been beneficial.

Differing individual participant abilities and suitability for the group environment were identified as barriers to implementation by many of the exercise facilitators, especially those facilitating larger group sizes.

*‘Let’s say one of the participants is less mobile, for example, the rest of the groups will also dial down for the participant. I mean they’re a very group culture, they’re a very beautiful group. But that also I feel sort of – you know, stops the progression.’* (Regional, NSW)

To address these barriers and help participants integrate into the group, the option for one-on-one sessions was recommended for future programming.

*‘Whereas if maybe they had like a separate one-on-one – and we could really dig down into what’s going on with them, we could say, okay, cool, this is what you’re going to do in the group class.’* (Regional, NSW)

### Processes of implementation

The appointment of an Aboriginal site manager was identified as a critical facilitator of the implementation process. The site manager played a pivotal role in bridging the gap and building trust between the community and the exercise facilitator, particularly as most of the exercise facilitators were not Aboriginal, or where the facilitator did not previously know the group. Other roles of the site manager included reminders and follow-ups for participants, scheduling additional health appointments and organising transport.

*‘Having (site manager) in as a conduit between us has been really helpful. Because then obviously they know him, they’re really comfortable with him. So having that introduction I think that’s been really helpful….. I’d say (for) attendance and buy-in, and with people feeling comfortable.’ (Regional, NSW)*

Secondly, data collection methods and the evaluation process were identified as factors affecting the implementation process. Many facilitators felt that having access to the data collected by the research team would have been beneficial for tailoring exercise prescriptions and monitoring progress.

*‘…Maybe (it) would have been a bit more interesting for us if we had been a bit more involved in the initial testing, to see where people were.’ (Regional, NSW)*

*‘It’d probably be interesting what a physical assessment was like at six months. “Cause sometimes it’s” better, then it gets worse again, and sometimes it’s just gradually improving.’ (Regional, NSW)*

### Intervention characteristics

Several intervention characteristics were identified as impacting implementation. Firstly, the emphasis on culture, kinship and a mob-led approach (where program activities and design were guided and shaped by the collective group of Aboriginal participants and Elders, rather than imposed by external facilitators) was a key intervention characteristic that facilitated implementation.

‘*This is a little bit of feedback from a few of the Elders, is that they have ownership of it. It’s their program. That was the really big change I saw between Ironbark and [another falls prevention program].’ (Regional NSW)*

The flexibility and adaptability of the program based on community needs and preferences were key facilitators across sites. All exercise facilitators discussed how they adapted the program to their group needs and valued the flexibility in the program design to allow this. They ensured core components, including the Otago exercises and Yarning components, were delivered, but the structure of the program, style of session (e.g. individual circuit versus group-based) and location were all varied based on community preferences.

*‘You have to be a little bit flexible too, because sometimes people wanted the exercise first. So if things weren’t ready, we’d do that. And so it just depended on, sometimes on the day, but generally, I just spoke to whoever the Aboriginal health worker was to see which suited them better.’ (Regional, NSW)*

*‘…especially at times of Sorry Business…there is a deep compassion…the group will provide guidance and support our understanding of how they would like to see things done and experience the Ironbark gathering, at certain times.’ (Urban, WA)*

Most facilitators reported that the exercise dose was suitable and acknowledged that, while the evidence recommends more sessions per week, this would likely not be feasible for participants or the services. However, some facilitators suggested the need for more supervised sessions and that compliance with the prescribed home programs was low.

*‘I would personally say that they’d need more than one per week. Some wouldn’t always do it at home and they would be just so honest with you that they needed that extra support and they felt so much better in that group setting.’ (Urban, NSW)*

The resources, including the basic exercise equipment and exercise booklets, which were provided to all sites, were reported as being suitable.

*‘Some Elders liked the laminated summary sheet of exercises. They could keep it on their fridge or on the kitchen bench as a reminder and it didn’t matter if it got wet or they spilt something on it.’ (Urban, WA)*

Another intervention characteristic that was identified was the program design and content. Both the exercises and Yarning topics became repetitive for many groups towards the end of the program.

*‘Yarning topics, trying to get a few more external talkers in probably would ultimately help… cause I think just reviewing all the same topics got a bit flat…We have had issues in the last three months just keeping motivation.’ (Regional, NSW)*

To maintain enjoyment and motivation they reported incorporating games, circuits, other cardiovascular exercise or upper body exercises to maintain enjoyment and motivation. One site discussed incorporating Aboriginal movement patterns into the exercise program, such as hunting or gathering movements or mimicking the movements of native animals.

The suitability of facilities emerged as factor in the implementation of the program. Facilitators discussed delivering the program across various settings, including community centres, health services and private exercise physiology clinics. The impact of the facility on program delivery varied by location. For instance, at one site, the small room size hindered the ability to run a circuit, limiting participants’ ability to progress individually. Conversely, locations within gyms allowed facilitators to utilise existing gym equipment beyond that provided by the research team.

*‘I haven’t used the bands, as much, just because we have a fully stocked gym, actually cables instead, but I heard participants using bands a lot at home.’ (Regional, NSW)*

Some expressed a preference for community-based settings to refer participants to other services;

*‘I actually think it’s really important to run it within the service if we can. Um, and that’s what I really liked (Site X) actually having it on site in the Aboriginal Health Centre, because it actually engaged them into the primary health care services as well. So it was a familiar place.’ (Regional, NSW)*

### Inner setting

Support from leadership within the employment structure, as well as from the research team, was a crucial factor for successful implementation. Participants noted that various employment structures existed, with some facilitators employed through the service and others as contractors. However, as long as support was provided, this variation did not seem to affect the delivery of the program. Some sites reported spending additional time on the program beyond the allocated hours to ensure Yarning topics were well planned and invited speakers were organised.

*‘But for me that supportive leadership has been important, because of that outside funding that we’ve got - - our leadership has been really supportive of us investing - - - you know, the extra time in and dedication to make the program what it is.’ (Urban, NSW)*

Another participant highlighted the practical support provided by local leadership,

*‘X and her team has been really good here in X council and they have been really kind to all our participants, you know, addressing their needs when it’s needed. For example, sometimes we forget to bring milk, they provide milk, and they are – you know, the room is always set up quite well.’ (Urban, WA)*

In addition to the support from the on-site leadership team, the online support and visits from the research team were highly valued. Receiving feedback was appreciated as part of the process evaluation coaching tool. Participants also valued learning about successful strategies from other sites and participating in occasional group check-ins to share ideas and best practices, such as warm-up activities and games.

*‘Having you guys coming out though initially and reviewing the class was excellent, just to give me feedback to make sure that I was doing the right thing for the program, but also as a professional.’ (Regional, NSW)*

Group dynamics were also identified as a significant factor. Overall, the groups and the social connections they provided were seen as key facilitators. However, some difficulties were reported by facilitators in managing louder voices during the Yarning sessions and navigating the dynamics between some group members.

*‘..some of our Elder women are sensitive to some of the humour by some of our men. That’s something that has had to be managed.’ (Urban, NSW)*

Another key facilitator suggested for the longer-term sustainability of the program was building capacity among the existing service staff. This involved supporting the group to run the program with less supervision. For example, training allied health assistants or Aboriginal Health Workers.

### Outer setting

Various environmental barriers to implementation were reported included floods, bushfires and COVID-19. Some sites were required to pause sessions due to these challenges for varying durations. Facilitators discussed the resilience of communities and the strategies they employed to maintain engagement, such as checking in with participants via phone and encouraging home exercises.

Securing funding for ongoing implementation was identified as a critical concern for the sustainability of the program.

*‘I guess the reality is, like I think of other things (important for sustainability), but then they come back to funding. You know, like, say, like, transport? But that comes back to funding. Like to some degree, supportive leadership (is important). But for me, that supportive leadership has been because of that outside funding that we’ve got.’ (Urban, NSW)*

While some sites reported success in obtaining funding, others were still in the process of seeking financial support. Key funding requirements for success included not only the facilitators’ time but also provisions for morning tea and transportation.

‘*I look across the sites what particularly makes that group easier is if you also have provided transport.’ (Urban, WA)*

The relationship between organisation and community was discussed as an important facilitator to implementation for community buy-in and trust. At sites where the facilitators were not familiar with participants in the group beforehand, this factor was particularly important.

*‘It was very, very helpful that I had met the other participants that, um, because in our culture – even though I’m an Indigenous person, first and foremost they have to, you know, trust you as a person.’ (Urban, NSW)*

### Implementation outcomes

For facilitators, the program provided valuable learning opportunities and capacity building, enhancing their professional skills and confidence. Additionally, facilitators improved their knowledge of cultural safety, which enabled them to deliver the program more effectively and sensitively within the community. They also gained insights from the community and its rich culture, which further enriched their understanding and approach.

*‘I thought the actual program became more than exercise--- I believe that people not only turn up for the exercise, but for the social part of it and the Yarn. I honestly think that is a big deal.’ (Regional, NSW)*

*‘I really discovered how important connection is and kinship and knowing that, if your mental health and your wellness and your happiness isn’t there, the physical health won’t follow, and that was probably one of the biggest things I took out of it all.’ (Regional, NSW)*

## Discussion

This study evaluated the implementation of Ironbark, a culturally tailored, multi-site falls prevention program for older Aboriginal people. Guided by the CFIR framework, key facilitators and barriers influencing program delivery across diverse community settings were identified. Program fidelity and dose were consistently high and positive implementation outcomes were reported. Community leadership, cultural relevance, supportive site management and program flexibility were perceived as critical enablers of engagement and adaptability without compromising core components. Barriers such as diverse participant abilities, content repetition, environmental disruptions and funding limitations were reported as common but often mitigated by strong organisational leadership, facilitator innovation and community resilience. Facilitators also reported professional growth and perceived that participants experienced enhanced social connection and wellbeing.

### Facilitators

The significance of community ownership was a theme that was common across different levels of the CFIR framework, including intervention characteristics, implementation process and the inner setting. This strategy has previously proven effective for improving health outcomes for Aboriginal people [28]. For example, at the intervention level, co-designing the intervention with community partners prior to implementation and ensuring the program was Aboriginal-led during implementation ensured the program met each sites unique needs. Community leadership in physical activity programs has previously been identified as a key enabler for participation among older Aboriginal people [29].

In addition, the program allowed for adaptations and flexibility in structure in response to participants’ preferences, which was discussed as a facilitator and was also observed in the site visit checklists. This flexibility was perceived to have led to high acceptability among sites and communities and was seen as contributing to sustained participation by allowing sites to tailor delivery to local needs while maintaining engagement. Despite differences in the delivery between sites, the core intervention components and dose were consistent, as assessed by the fidelity checklists. Within the implementation process domain, the employment of an Aboriginal site manager was another facilitator discussed, again promoting community ownership, trust-building and participant engagement.

Another theme that extended across the inner and outer setting factors was the perceived importance of supportive leadership. Buy-in and support from leaders within the services delivering the program were discussed as an essential factor for successful implementation and a facilitator for future sustainability and funding. A review of barriers and facilitators to implementation of community-based physical activity programs also identified leadership as a key facilitator [30]. In organisations where existing projects were extended, support from project leaders promoted sustainability. Leaders also served as role models within their organisations, facilitating implementation and championing physical health initiatives.

### Barriers

Several barriers to implementation were discussed, including difficulties with group dynamics and differing individual physical abilities, which may have hindered the progress of some participants. Nevertheless, facilitators reported that social benefits were often prioritised by the group. Some exercise professionals noted that the program became repetitive and boring for participants, which reflects previous research indicating that older adults prefer exercises that are varied, engaging and enjoyable [31]. Therefore, it is recommended to provide more comprehensive guidance and resources to encourage and support facilitators in developing creative ideas to diversify the program over the 12-months. For example, the development of a content calendar with the inclusion of rotating guest speakers. In addition, reasons for lower attendance or fidelity were explained in the open text sections and reinforced by the qualitative interviews, commonly at the outer and inner setting levels, including environmental barriers, cultural events and staff and venue changes. Facilitators highlighted the resilience of the community participants and sites to adapt to changing circumstances.

Training and confidence of the exercise facilitators varied, which may have impacted the quality of the delivery. However, training conducted by the research team and ongoing support were valued. It is important to reflect on how this could be done as Ironbark and other community-based physical activity programs move from research team-supported to sustainability. A process evaluation was conducted for a physical activity program for Indigenous women in Australia and explained the importance of investing resources to develop a professional program that offers consistent training, technical support and feedback [32]. This may be particularly important for the sustainability of falls prevention programs for older Aboriginal People.

### Sustainability

A key barrier to the sustainability of the program was ongoing funding. This is critical to address, given cost is a key barrier to participation for older Aboriginal people [29]. To address this, Ironbark facilitators proposed strategies such as building capacity among local staff, including Aboriginal Health Workers and reducing the frequency of visits from physiotherapists or exercise physiologists for program adjustments and progress tracking. It is also important to acknowledge that facilitators’ emphasis on funding sustainability may be influenced by their professional investment in the program and its continuation, which should be considered when interpreting these findings.

Aboriginal leaders continue to advocate for increased funding for Aboriginal Community Controlled Health Organisations (ACCHOs) to deliver culturally safe and community-driven programs. Ironbark aligns with this model, with previous studies demonstrating its feasibility and effectiveness for older Aboriginal people and the current findings further highlighting how delivery within trusted community settings supports engagement and implementation. While long-term sustainability and funding models were not directly examined in this study, previous research has shown that publicly funded programs are more likely to be sustainable when delivered through ACCHOs, as these places are supported by older Aboriginal people [33, 34].

Previous researchers have developed a guide for planning for sustainability and scale-up of physical activity interventions [17]. The guide focuses on (i) identifying differences between the research and practice context that can be addressed during intervention development and implementation planning by focusing on system, delivery personnel and intervention characteristics; and (ii) early planning for implementation barriers and facilitators. This includes who, what and how the intervention would be delivered if research funding has ceased.

### Limitations and future research

The study had several limitations. Firstly, facilitators were aware of site visits, which may have influenced the delivery of observed sessions. These observations were also conducted by research team project officers, which may have introduced potential observer or social desirability bias and should be considered when interpreting the findings. The data collected also did not allow for a quantitative examination of the relationship between the identified implementation factors and the implementation scores derived from the site visit data. Future studies might explore which types of training and technical support are most beneficial to exercise facilitators and other program leaders to enhance their capacity to navigate factors that can interfere with program implementation.

Furthermore, this study focuses on facilitator perspectives of program delivery and implementation. Although Aboriginal community and participant perspectives were collected as part of the broader Ironbark study, these findings are reported separately, limiting integration of participant experiences within this paper. Lastly, we do not have implementation data for one site, which was delivered intermittently due to outer-level barriers, including floods, COVID-19, and individual-level barriers, including sickness and family responsibilities. Similar outer-level barriers and travel restrictions also led to missing data at other sites.

## Conclusion

These findings highlight the feasibility of implementing a community-led fall prevention program for older Aboriginal people across community settings. High program fidelity was achieved, supported by community leadership, flexible delivery and strong organisational engagement. The findings highlight that sustaining programs like Ironbark would likely require dedicated funding, local workforce capacity and mechanisms to support independent delivery and evaluation. This process evaluation offers practical guidance for embedding culturally safe, strength-based health programs for Aboriginal communities and considerations to inform broader implementation efforts across other Indigenous and underserved communities.

## Supplementary Material

Supplementary_materials_afag184

## Ironbark Investigator Group

Ironbark Investigator Group: Kathie Clapham, Lindy Clemson, Robert Cumming, Margaret Gidgup, Josephine Gwynn, Kirsten Howard, Kate Hunter, Stephen Jan, Marion Kickett, Lisa Keay, Cassia Lindsay, Tamara Mackean, Kris Rogers, Judy Simpson, Amy Townsend.
