# Supplementary material for: Implementation of the Ironbark falls prevention program: a mixed methods process evaluation with Aboriginal communities
Source: Age Ageing. 2026 Jun 22;55(6):afag184. doi: 10.1093/ageing/afag184 (PMC13284708; doi:10.1093/ageing/afag184)
Supplement: Supplementary_materials_afag184 [file supplementary_materials_afag184.docx]

**Supplementary Material**

***Appendix 1****. Exercise facilitator interview questions*

- Can you firstly tell me about your professional background?
- Did you have any experience in falls prevention before the Ironbark program? Have you worked with older Aboriginal people before Ironbark?
- How were you employed for the Ironbark project? E.g., as an external contractor or embedded within the AMS? How did you find that worked?
- First question – I want to know whether you were satisfied with how the falls prevention program was delivered? Why/why not?
- Were you satisfied with the support provided by the research team? How could this be improved for future groups?
- How did the program fit within the culture and structure of the organisation? Was the organisation supportive of the program?
- Were you satisfied with the dose of the program the participants received?
- What are your thoughts on the structure of the intervention and resources provided? Is there anything you would change?
- Did you need to adapt or tailor the program to meet your group’s needs?
- Were there any other contextual factors that affected the implementation of the program e.g., environmental, political, social?
- What components of the program do you think are important for the sustainability of the program?

***Appendix 2.*** *Missing process evaluation data*

|  | **Checklist** |  |  | **Program delivery** |  |  | **Interviews** |
| --- | --- | --- | --- | --- | --- | --- | --- |
| Site | 8 week | 6 month | 9 month | 8 weeks | 6 month | 9 month |  |
| 3 |  |  |  |  |  |  |  |
| 4 |  |  |  |  |  |  |  |
| 6 |  |  |  |  |  |  |  |
| 4 |  |  |  |  |  |  |  |
| 8 |  |  |  |  |  |  |  |
| 9 |  |  |  |  |  |  |  |
| 10 |  |  |  |  |  |  |  |
| 11 |  |  |  |  |  |  |  |
| 12 |  |  |  |  |  |  |  |
| 13 |  |  |  |  |  |  |  |
| 14 |  |  |  |  |  |  |  |
| 18 |  |  |  |  |  |  |  |
| 19 |  |  |  |  |  |  |  |
| 20 |  |  |  |  |  |  |  |
| 21 |  |  |  |  |  |  |  |
|  |  |  |  |  |  |  |  |
|  |  |  |  |  |  |  |  |
|  |  | Missing |  |  |  |  |  |
|  |  | Complete |  |  |  |  |  |

| **Appendix 3. Program checklist and coaching tool** | | | |
| --- | --- | --- | --- |
| Site: Facilitator: | Completed by: Program week number: | | |
| **Key element** | | **Completed** | **Comments** |
| **1. Introduction** | | | •Do a general unstructured yarn (check in/welcome) with participants?  •Describe the mood of the room? (calm, excited, busy, tense, is there nervous laughter, is there awkward silence?) |
| Participants welcomed | | **Y / N** |  |
| Purpose and aims of program explained | | **Y / N** |  |
| **2. The facilitator explained the following key points of the Program:** | | |  |
| Preventing falls | | **Y / N** |  |
| Embedding strength & balance activities in everyday life | | **Y / N** |  |
| Looking for opportunities to practice | | **Y / N** |  |
| Modify environment to increase challenge | | **Y / N** |  |
| Challenging yourself (no set number) | | **Y / N** |  |
| Being safe | | **Y / N** |  |
| **3. The session activities included:** | | | |
| Recap of how to improve ***strength*** e.g. increase reps, do different exercises, use weights/bands | | **Y / N** | •Do the participants appear happy and comfortable  •Listen to what is being said. Note language / words being used  •Note the ways in which participants and facilitators interact?  •How do participants interact with each other?  •Is the facility / equipment suitable?  •Are there additional Aboriginal cultural components that we haven’t captured elsewhere? |
| Recap of how to improve ***balance*** e.g. increase reps, reduce your hand support, hold for longer | | **Y / N** |  |
| Yarning circle for participants | | **Y / N** |  |
| Facilitator and participant choosing exercises together | | **Y / N** |  |
| Facilitator demonstrates the Ironbark exercises | | **Y / N** |  |
| Participants performs new and existing exercises | | **Y / N** |  |
| Facilitator refers to **Ironbark Manual** (if appropriate) to explain and reinforce the exercises | | **Y / N** |  |
| Facilitator explains home exercises | | **Y / N** |  |
| **7. Overall** | | |  |
| Participants are engaged in exercise program | | **Y / N** |  |
| Participants are engaged in the yarning circle | | **Y / N** |  |
| Participants ask facilitator questions | | **Y / N** |  |
| Site manager & facilitator appear to be working well together | | **Y / N** |  |
| Session facilitated in a culturally appropriate way | | **Y / N** |  |
| Facilitator respectful of all participants | | **Y / N** |  |

Additional comments you would like to tell us about the session today/any suggestions you would like to make:

____________________________________________________________________________________________________________________________________________________________________________________________________________________________________________________________________________________________________________________________________________________________________________________________________________________________________________________________________________________________________________________________________________________________________________________________________________________________________________________________________________________________________________________________________________________________________________________________________________

**Appendix 4. Program Delivery** **(dose delivered) – to what extent were all the intended program components delivered to participants?** (to be completed by Facilitators at 2, 6, 9, 12 months)

Please answer the following questions *about the most recent weekly Ironbark program you delivered*. Once completed, email this document back to the project officer.

1. Which site are you from? _______________ 2. State: ☐ NSW ☐ SA ☐ WA

3. Date of most recent weekly program __ / __ / __

4. Did the session go according to your plan? ☐ Yes ☐ No ☐ Unsure

5. If not, did you still meet some/all of the intended objectives of the session?

☐ Yes ☐ No ☐ Unsure ☐ N/A

5.How long was the yarning session?

Under 30 minutes ☐ 30 - 45 minutes ☐ 45 – 60 minutes ☐ Over 60 minutes ☐

6. How involved was the group in the yarning session?

| 1 | 2 | 3 | 4 | 5 |
| --- | --- | --- | --- | --- |
| Not involved |  |  |  | Very involved |

7. From your observations, how relevant/interesting was todays yarning topic to your group?

| 1 | 2 | 3 | 4 | 5 |
| --- | --- | --- | --- | --- |
| Not relevant |  |  |  | Very relevant |

8.How long was the exercise session?

Under 30 minutes ☐ 30 - 45 minutes ☐ 45 – 60 minutes ☐ Over 60 minutes ☐

9. How involved was the group in the exercise session? (please circle a number)

| 1 | 2 | 3 | 4 | 5 |
| --- | --- | --- | --- | --- |
| Not involved |  |  |  | Very involved |

10. Did you modify the program to meet individual participant needs (e.g. mobility)?

☐ Yes ☐ No ☐ Unsure ☐ N/A

11. Did you correct participant techniques, as needed?

☐ Yes ☐ No ☐ Unsure ☐ N/A

12. How important was the exercise equipment provided (e.g. balance boards) to the session?

| 1 | 2 | 3 | 4 | 5 |
| --- | --- | --- | --- | --- |
| Not important |  |  |  | Very important |

13. Did you explain home exercises to the participants?

☐ Yes ☐ No ☐ Unsure ☐ N/A

14. Did you deliver an appropriate **number** and **level** of exercises for all participant abilities?

☐ Yes ☐ No ☐ Unsure ☐ N/A

Additional comments you would like to tell us about the session today/any suggestions you would like to make:

_______________________________________________________________________________________________________________________________________________________________________________________________________________________________________________________________________________________________________________________________________________________________________________________

_______________________________________________________________________________________________________________________________________________________________________________________________________________________________________________________________________________________________________________________________________________________________________________________

_______________________________________________________________________________________________________________________________________________________________________________________________________________________________________________________________________________________________________________________________________________________________________________________

_______________________________________________________________________________________________________________________________________________________________________________________________________________________________________________________________________________________________________________________________________________________________________________________
